# Nomogram for Prediction of Bronchial Mucus Plugs in Children with *Mycoplasma pneumoniae* Pneumonia

**DOI:** 10.1038/s41598-020-61348-w

**Published:** 2020-03-12

**Authors:** Xuefeng Xu, Huiwen Li, Yuanjian Sheng, Lei Wu, Danli Wang, Lingyue Liu, Yu Tong, Zhimin Chen

**Affiliations:** 1 Department of Respiratory Medicine, Zhejiang University School of Medicine, National Clinical Research Center for Child Health, Hangzhou, 310052 China; 2 Department of Rheumatology Immunology & Allergy, The Children’s Hospital, Zhejiang University School of Medicine, National Clinical Research Center for Child Health, Hangzhou, 310052 China

**Keywords:** Bacterial infection, Paediatric research, Risk factors

## Abstract

The presence of bronchial mucus plugs (BMP) in children with *Mycoplasma pneumoniae pneumonia* (MPP) results in delayed clinical and radiographic resolution and long-standing pulmonary sequelae. The predictive factors associated with BMP formation remains poorly defined. Nomograms to predict BMP presence in children with MPP were proposed using a cohort of patients who underwent bronchoscopy intervention at Children’s Hospital in Eastern China. Patients with MPP in an earlier period formed the training cohort (n = 872) for nomogram development, and those thereafter formed the validation cohort (n = 399) to confirmed model’s performance. BMP in children with MPP were found in 196 (22.5%) and 91(22.8%) patients in the training and validation cohorts, respectively. The independent risk factors associated with BMP were age >5years (OR 2.06; 95% CI 1.43 to 2.98), higher IL-10 level (>10 ng/L, 2.19; 95% CI 1.46 to 3.28), higher IFN-γ level (>30 ng/L, 1.69; 95% CI 1.13 to 2.54), and presence of complication (3.43; 95% CI 1.45 to 8.09). Incorporating these 4 factors, the nomogram achieved good concordance indexes of 0.771(95% CI, 0.734–0.808) and 0.796 (95% CI, 0.744–0.848) in predicting BMP in the training and validation cohorts, respectively. The nomogram achieved an optimal prediction of BMP in children with MPP. Using this model, the risk of BMP formation would be determined, contributing to a rational therapeutic choice.

## Introduction

Community-acquired pneumonia (CAP) is a significant cause of morbidity and mortality worldwide and a major public health threat to children in China^1^. More than one fourth of children in the developing countries will have an episode of pneumonia during the first 5 years of life and there are about 2 million deaths per year^2,3^. *Mycoplasma pneumoniae* (*M. pneumoniae*) is one of the main pathogens causing CAP in young adults and children^4^. *M. pneumoniae* pneumonia (MPP) accounts for up to 40% or more of CAP in children, increasing the rate of morbidity, mortality, as well as the cost of health care in China^5–8^. Although MPP is usually a benign and self-limited disease, a number of cases had been reported to develop into refractory MPP^5,9–12^. Furthermore, more than a third of refractory MPP were found to have bronchial mucus plugs (BMP) formation^9^. Refractory MPP children with BMP presented with delayed clinical and radiographic resolution and long-standing pulmonary sequelae such as bronchiectasis and atelectasis, even causing plastic bronchitis or requiring extracorporeal membrane oxygenation^5,9,13,14^. Early bronchoscopy intervention contributed to removing BMP and reducing the risk of pulmonary sequelae. Furthermore, bronchoscopy combined with BAL has an important role for diagnosis in the evaluation of airway abnormality and pulmonary infiltrates in children, in whom rapid and accurate diagnosis is crucial for survival^15^. Therefore, an accurate preoperative estimation of BMP presence in children with MPP would help pediatricians perform bronchoscopy intervention, possibly leading to decreased pulmonary sequelae.

The formation of BMP could be associated with inflammatory factors, oxidants, and immune reaction^5,12,16,17^. A study by Xu *et al*. showed that age, fever duration, C-reactive protein (CRP) and lactic dehydrogenase (LDH) as independent risk factors for BMP presence in children with MPP^5^. Another study revealed that the combination of age and serum levels of LDH, erythrocyte sedimentation rate (ESR), and CRP might be a predictive panel marker for early prediction of refractory MPP^18^. Additionally, there is evidence that increased serum concentrations of tumor necrosis factor (TNF) alpha, interferon (IFN) gamma, and interleukin 18 (IL-18) were closely associated with refractory MPP^19–21^. Although atelectasis is a stronger indicator for bronchoscopy intervention, many MPP children with BMP had no significant atelectasis signs in chest imaging. Thus it is especially essential to predict BMP presence in children with MPP using some key clinical variables. Therefore, the aim of the present study was to define clinical factors associated with BMP formation in MPP children using a cohort of patients. In particular, we sought to create an internally validated nomogram to predict the individual risk of BMP in MPP children.

## Methods

### Patients and data collection

A retrospective study was conducted on a primary cohort of patients who had MPP and underwent flexible bronchoscopy between January 2016 and December 2018 at the respiratory department of Children’s hospital, Zhejiang University School of Medicine (Hangzhou, China). Diagnosis of MPP was based on diagnosis of CAP and etiology using our previous study^22^. Those patients with tuberculosis or HIV positive were excluded from the study, and those with chronic lung diseases, congenital heart disease, cerebral palsy or tumor were also excluded. Eligible consecutive patients with MPP between January 2016 and December 2017 were included into the training cohort for development of the nomogram to predict risk of BMP, and those between January and December 2018 were enter into the validation cohort.

Demographic and clinical data were collected, including imaging examination (suggestive of pneumonia or pulmonary consolidation, atelectasis and pleural effusions), ultrasonography (suggestive of pleural effusions), and bronchoscopy results. All laboratory tests for collection were performed before bronchoscopy. Based on our previous study, the cutoff values of IL-10 and IFN-γ were determined as 10 ng/L and 30 ng/L, respectively^22^. The definition of complications referred to the presence of pulmonary atelectasis and/or pleural effusion. The primary outcomes of interest were risk of BMP formation in children with MPP.

### Ethical considerations

This study was approved by the Ethic Review Board of Children’s Hospital, Zhejiang University School of Medicine. Written informed consents were obtained from a parent and/or legal guardian of each participant and all research was performed in accordance with the relevant guidelines and regulations.

### Statistical analysis

Continuous variables were expressed as mean and standard deviation (SD), and compared using an unpaired, 2-tailed t test or Mann-Whitney test. Categorical variables were compared using X^2^ test or Fisher exact test. Clinical variables associated with increased risk of BMP formation were assessed based on clinical importance, scientific knowledge, and predictors identified in previously published articles^22–24^. The associations of relevant clinical variables with BMP formation in MPP children were assessed using logistic regression models. Stepwise analysis with the Akaike information criterion (AIC) was used to identify variables for the multivariable regression models. Odds ratios (OR) were reported with their 95% CIs. Selected variables were incorporated in the nomogram to predict the probability of BMP formation in MPP children using statistical software (*rms* package of R, version 3.4.3; http://www.r-project.org). The nomogram was based on regression coefficient in multivariate logistic regression to 0 to 100-point scale.

The model performance was evaluated by the concordance index (C statistics) and calibration. The C statistic estimates the probability of concordance between predicted and observed outcomes and is equivalent to the area under the receiver operating characteristic curve (ROC). ROC analysis was used to calculate the optimal cutoff value, determined by maximizing the Youden index. Accuracy of cutoff value was assessed by the sensitivity, specificity, and predictive values. Calibration was evaluated using a calibration plot, a graphic representation of the relationship between the observed outcome and the predicted probabilities. The model was validated using bootstrapped resampling to decrease the overfitting bias^25,26^. R statistical software packages were used to perform all the statistical analysis and graphics (R code, see supplementary information), P < 0.05 was considered statistically significant.

## Results

### Clinical characteristics

**Figure 1:**
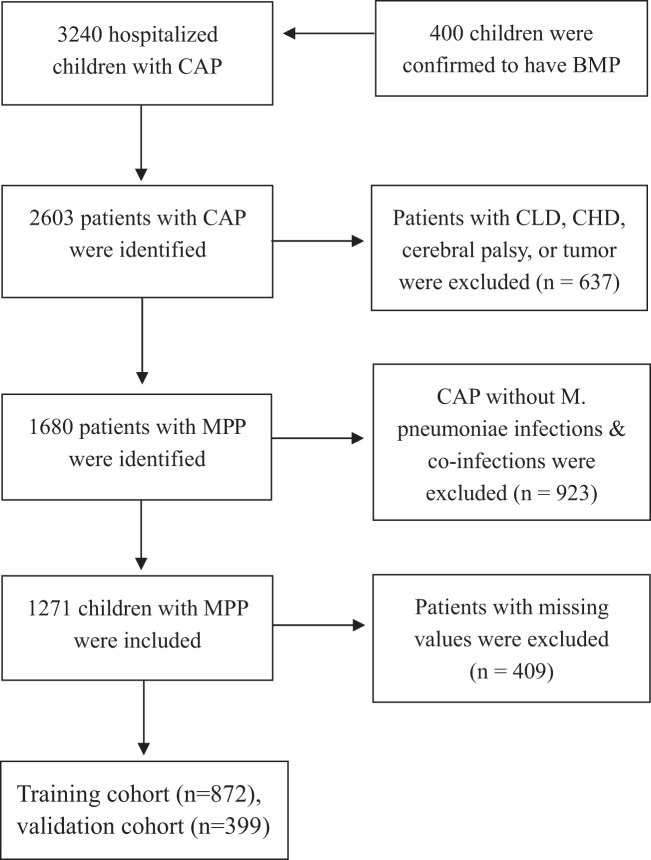
Study flow. BMP = bronchial mucus plugs; CAP = community-acquired pneumonia; CLD = chronic lung disease; CHD = congenital heart disease; MPP = *Mycoplasma pneumoniae* pneumonia.

**Figure 2:**
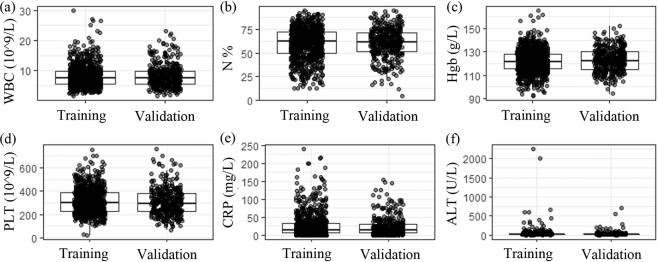
Distribution of clinical characteristics between the training and validation cohorts. (**a**,**b**,**c**,**d**,**e**, and **f**) represented white blood Cell (WBC) counts, neutrophil percentage (N%), hemoglobin (Hgb) concentration, platelet (PLT) counts, C reactive protein (CRP) and alanine aminotransferase (ALT) levels, respectively.

**Figure 3:**
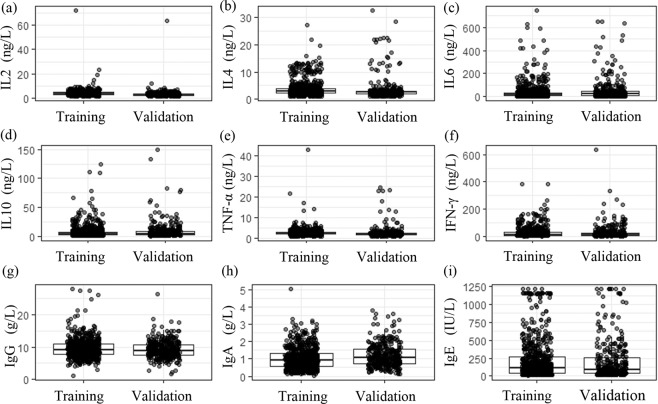
Comparisons of clinical characteristics between the training and validation cohorts. The dot plot showed cytokine and immunoglobulin levels, respectively.

Four hundred children undergoing bronchoscopy were confirmed to have bronchial mucus plugs (BMP). Patients who had no *M. pneumoniae* infections and those with missing values on relevant predictors were not included in the study. Therefore, 287 children with MPP and BMP were included in the analytic cohort. Totally, 1271 children with MPP met the inclusion criteria. 872 and 399 children with MPP were divided into the training and validation cohorts, respectively (Fig. 1). The clinical characteristics of patients are seen Table 1. The baseline clinical data were similar between the training and validation cohorts, except serum IgA and IL-6 levels (Figs. 2 and 3). Bronchoscopy identified BMP in children with MPP were found in 196 (22.5%) and 91(22.8%) patients in the two cohorts, respectively.

**Table 1:** Clinical characteristics of patients in the training and validation cohort.

Variable	Training cohort (n = 872)	Validation cohort (n = 399)	P value
Plugs (Y/N)	196/676	91/308	0.90
Gender (M/F)	481/391	209/190	0.36
Age (year)	5.20 ± 2.93	4.99 ± 2.86	0.20
Age (>5 y, n)	387	165	0.31
Birthweight	3.30 ± 0.49	3.32 ± 0.51	0.307
Fever (day)	8.64 ± 4.41	8.77 ± 5.32	0.85
WBC counts (x10^9/L)	8.21 ± 3.65	8.31 ± 3.80	0.65
Neutrophil (%)	60.08 ± 16.66	60.89 ± 15.57	0.41
Hgb (g/L)	121.940 ± 10.38	122.79 ± 9.74	0.17
PLT (x10^9/L)	312.62 ± 108.12	306.35 ± 114.13	0.35
CRP (mg/L)	26.63 ± 33.75	23.60 ± 26.47	0.08
PCT (ng/L)	0.35 ± 2.07	0.27 ± 0.52	0.54
ALT (U/L)	31.90 ± 112.67	25.62 ± 52.57	0.29
AST (U/L)	51.34 ± 151.55	45.87 ± 61.16	0.49
IgG (g/L)	9.51 ± 2.94	9.20 ± 2.71	0.09
IgA (g/L)	1.03 ± 0.64	1.19 ± 0.66	0.001**
IgE (U/L)	223.70 ± 285.99	207.38 ± 280.46	0.38
IL-2 (1.1–9.8 ng/L)	3.88 ± 2.88	3.19 ± 3.31	
IL-4 (0.1–3.0 ng/L)	3.74 ± 2.77	3.38 ± 3.86	0.06
IL-6 (1.7–16.6 ng/L)	36.69 ± 69.10	49.97 ± 90.46	0.009*
IL-10 (2.6–4.9 ng/L)	7.98 ± 10.19	9.27 ± 13.91	0.09
TNF-α (0.1–5.2 ng/L)	2.67 ± 1.93	2.55 ± 2.63	
IFN-γ (1.6–17.3 ng/L)	23.83 ± 36.51	25.28 ± 48.26	0.55
Pleural effusion (n)	244	112	0.53
Atelectasis (n)	124	63	0.93
Complications (n)	298	141	0.97

ALT = alanine aminotransferase; AST = aspartate aminotransferase; CRP = C reactive protein; ESR = erythrocyte sedimentation rate; Hgb = hemoglobin; IFN = interferon; IgA = Immunoglobulin A; IgG = Immunoglobulin G; IL = interleukin; PCT = procalcitonin; PLT = blood platelet; TNF = tumor necrosis factor; WBC = white blood cells. *P < 0.05, **P < 0.01.

### Predictors of BMP formation in children with MPP

**Figure 4:**
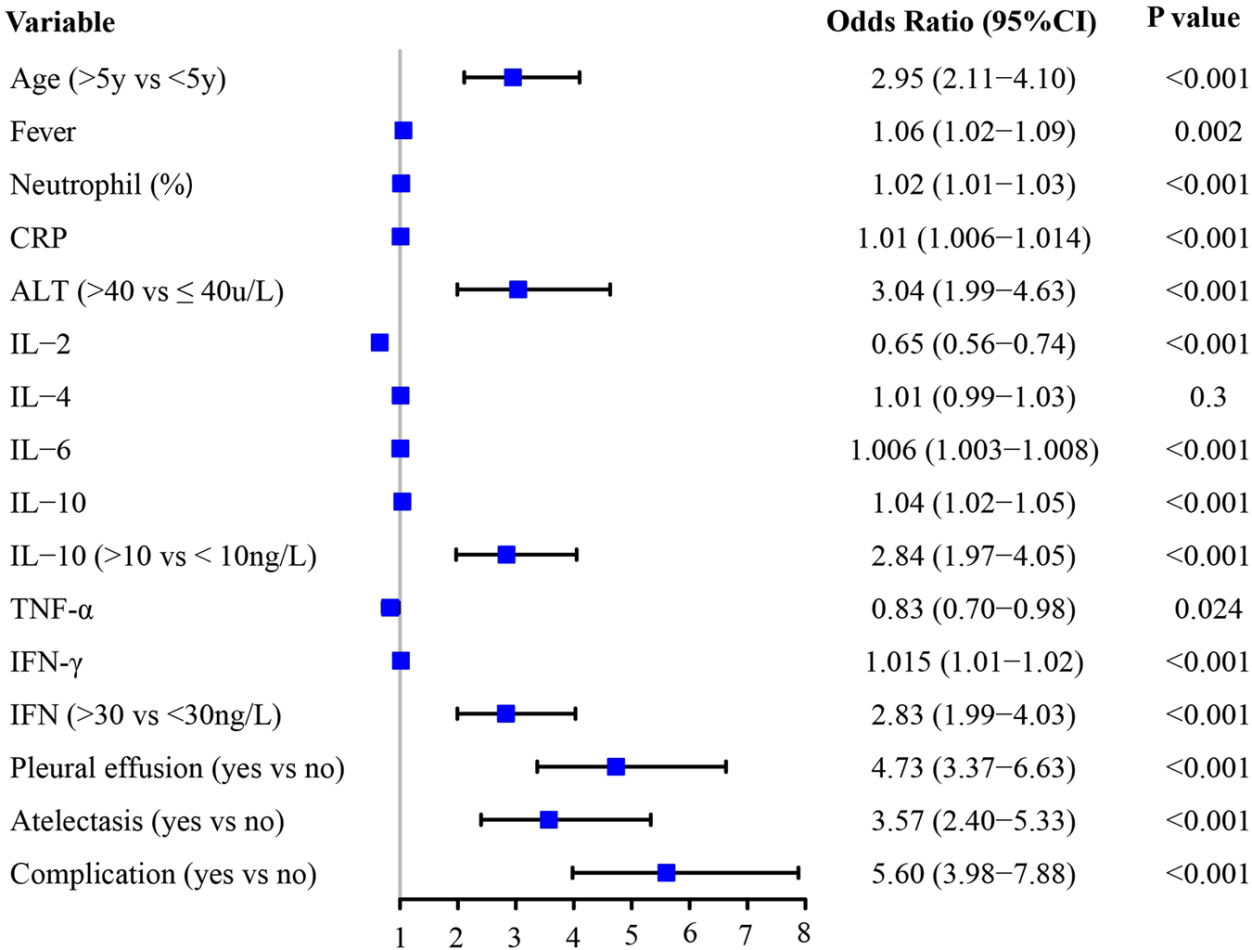
Odds ratio (OR) for the independent risk factors based on the training cohort. Univariate logistic regression analysis showed significantly higher OR of age >5 years, ALT > 40 U/L, IL-10 > 10 ng/L, IFN-γ > 30 ng/L, and the presence of complication. Complication referred to the presence of pulmonary atelectasis and/or pleural effusion.

In the training cohort, MPP children with BMP had longer duration of fever (7.18d vs 6.12d), higher neutrophil percentage (64.65% vs 58.75%), higher CRP level (36.64 vs 23.73 mg/L), higher IL-6 (61.25 vs 29.57 ng/L), IL-10 (11.28 vs 7.02 ng/L), and IFN-γ (40.58 vs 18.97 ng/L) levels compared with those without BMP (Table 2). A univariate logistic regression showed that age older than 5 years (OR, 2.95; 95% CI 2.11 to 4.10), longer fever time (OR, 1.06; 95% CI 1.02 to 1.09), higher neutrophil percentage (OR, 1.02; 95% CI 1.01 to 1.03), higher CRP level (OR, 1.01; 95% CI 1.006 to 1.014)), higher ALT (>40 vs ≤40 u/L; OR, 3.04; 95% CI 1.99 to 4.63), higher IL-6 (OR, 1.006; 95% CI 1.003 to 1.008), IL-10 (OR, 1.04; 95% CI 1.02 to 1.05), and IFN-γ (OR, 1.015; 95% CI 1.01 to 1.02) levels, presences of pleural effusion (OR, 4.73; 95% CI 3.37 to 6.63) and atelectasis (OR, 3.57; 95% CI 2.40 to 5.33) were independently associated with BMP formation in MPP children, especially higher IL-10 (>10 vs ≤10 ng/L; OR, 2.84; 95% CI 1.97 to 4.05), higher IFN-γ (>30 vs ≤30 ng/L; OR, 2.83; 95% CI 1.99 to 4.03), and presence of complication (OR, 5.60; 95% CI 3.98 to 7.88) (Fig. 4). On multivariate regression analysis, age >5 years (OR 2.06; 95% CI 1.43 to 2.98), higher IL-10 level (>10 vs ≤10 ng/L; OR, 2.19; 95% CI 1.46 to 3.28), higher IFN-γ level (>30 vs ≤30 ng/L; OR, 1.69; 95% CI 1.13 to 2.54), and presence of complication (OR, 3.43; 95% CI 1.45 to 8.09) were each independently associated with BMP formation (Table 3).

**Table 2:** Clinical characteristics of patients in the training cohort.

Variable	No plug (n = 676)	Plugs (n = 196)	P value
Birthweight	3.28 ± 0.50	3.35 ± 0.45	0.09
Fever (d)	6.12 ± 4.24	7.18 ± 4.25	0.002**
Gender (M/F)	378/298	103/93	0.40
Age (>5 y, n)	260	127	<0.01**
WBC counts (x10^9/L)	8.30 ± 3.73	7.90 ± 3.37	0.17
Neutrophil (%)	58.75 ± 16.60	64.65 ± 16.07	<0.01**
Hgb (g/L)	121.90 ± 10.45	122.07 ± 10.20	0.84
PLT (x10^9/L)	313.97 ± 106.11	308.01 ± 114.86	0.50
CRP (mg/L)	23.73 ± 27.25	36.64 ± 48.85	<0.01**
PCT (ng/L)	0.30 ± 2.14	0.50 ± 1.83	0.27
ALT (U/L)	30.01 ± 121.05	38.39 ± 76.98	0.36
AST (U/L)	49.44 ± 168.51	57.86 ± 65.22	0.50
LDH (U/L)	296.79 ± 124.84	311.92 ± 107.07	0.124
IgG (g/L)	9.50 ± 2.84	9.58 ± 3.27	0.76
IgA (g/L)	0.95 ± 0.61	1.29 ± 0.66	<0.01**
IgE (U/L)	231.01 ± 292.34	197.57 ± 261.25	0.17
IL-2 (ng/L)	4.08 ± 3.18	3.19 ± 1.23	<0.01**
IL-4 (ng/L)	3.79 ± 2.61	3.58 ± 3.25	0.41
IL-6 (ng/L)	29.57 ± 53.67	61.25 ± 102.84	<0.01**
IL-10 (ng/L)	7.02 ± 8.24	11.28 ± 14.65	<0.01**
TNF-α (ng/L)	2.73 ± 2.11	2.43 ± 1.12	0.05
IFN-γ (ng/L)	18.97 ± 26.81	40.58 ± 55.70	<0.01**
Pleural effusion (n)	137	107	<0.01**
Atelectasis (n)	68	56	<0.01**
Complications	170	128	<0.01**
ALT (>40 U/L)	62	46	<0.01**
IL10 (>10 ng/L)	105	67	<0.01**
IFN-γ (>30 ng/L)	113	71	<0.01**

ALT = alanine aminotransferase; AST = aspartate aminotransferase; CRP = C reactive protein; ESR = erythrocyte sedimentation rate; Hgb = hemoglobin; IFN = interferon; IgA = Immunoglobulin A; IgG = Immunoglobulin G; IL = interleukin; LDH = lactate dehydrogenase; PCT = procalcitonin; PLT = blood platelet; TNF = tumor necrosis factor; WBC = white blood cells. *P < 0.05, **P < 0.01.

**Table 3:** Multivariate logistic regression analysis of bronchial mucus plugs based on the training cohort.

Variable	Odds Ratio (95%CI)	P value
Age (>5 y vs ≤5 y)	2.06 (1.43–2.98)	<0.001**
ALT (>40 vs ≤40 U/L)	1.56 (0.97–2.57)	0.07
IL-10 (>10 vs ≤10 ng/L)	2.19 (1.46–3.28)	<0.001**
IFN-γ (>30 vs ≤30 ng/L)	1.69 (1.13–2.54)	0.011*
Pleural effusion (yes vs no)	0.97 (0.45–2.08)	0.93
Atelectasis (yes vs no)	1.41 (0.78–2.53)	0.25
Complications	3.43 (1.45–8.09)	0.005**

ALT = alanine aminotransferase; IL = interleukin; IFN = interferon. *P < 0.05, **P < 0.01.

### Development and validation of a BMP-predicted nomogram

**Figure 5:**
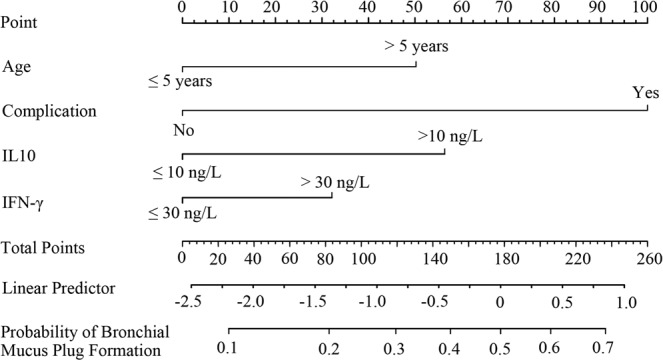
Nomogram to estimate the risk of BMP presence in MPP children. The nomogram to predict BMP was created based on 4 independent risk factors (age >5 years, higher IL10 level, higher IFN-γ level, and presence of complication). Points are assigned for each variable by drawing a line upward from the corresponding variable to the Points line. The sum of points plotted on the total Points line corresponds with the BMP probabilities in MPP children. Complication referred to the presence of pulmonary atelectasis and/or pleural effusion.

**Figure 6:**
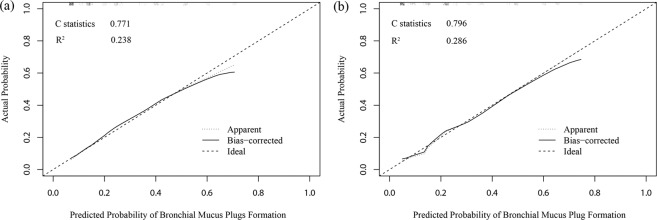
The bootstrapped estimates of calibration accuracy for the nomogram at the training (**a**) and validation cohorts (**b**). The ideal outcome (dashed line), the observed outcome (apparent, fine dashed line), and the bias-corrected outcome (solid line) are depicted.

Nomogram to predict BMP formation in children with MPP from the training cohort was shown in Fig. 5. The nomogram to predict BMP was created based on the following 4 independent predictive factors: age >5 years, higher IL-10 level (>10 vs ≤10 ng/L), higher IFN-γ level (>30 vs ≤30 ng/L), and presence of complication. Higher total points based on the sum of the assigned number of points for each factor in this nomogram were associated with the risk of BMP. The model demonstrated a good discriminative ability in estimating the risk of BMP, with a C statistics of 0.771 (95% CI, 0.734–0.808). Furthermore, calibration plot graphically showed a good agreement on BMP formation in children with MPP via bootstrap resampling (Fig. 6a).

In the validation cohort, the nomogram also showed a good discriminative ability for the estimation of BMP formation with C statistics of 0.796 (95% CI, 0.744–0.848). There was also a good calibration curve for the risk estimation of BMP in children with MPP (Fig. 6b). The optimal cutoff value of the total nomogram scores was determined as 80. The sensitivity and specificity when used in discriminating the presence of BMP from MPP children were 73.0% (95% CI, 70.0–75.9%) and 70.6% (95% CI, 67.6–73.6%) in the training cohort, and 84.6% (95% CI, 81.1– 88.1%) and 60.7% (95% CI, 58.3–63.1%) in the validation cohort, respectively.

## Discussion

The presence of BMP results in the poor clinical prognosis in children with MPP. Our study suggests that clinical variables, including age >5 years, higher IL-10 level (>10 ng/L), higher IFN-γ level (>30 ng/L), and presence of complication, are significantly associated with presence of BMP in children with MPP. Particularly, the presences of complications, including pleural effusion and atelectasis, strongly predicted the BMP formation in children with MPP.

In the present study, a univariate logistic regression showed that fever, neutrophil percentage, serum CRP, IL-2, IL-4, IL-6, and TNF-α levels were significantly associated with BMP formation in children with MPP. However, their OR values were relatively small, and had only a low statistical power. Therefore, these clinical variables were not included in further analysis. Additionally, multivariate regression model demonstrated that elevated serum ALT, the presence of pleural effusion or atelectasis had no significant correlation with BMP formation. Eventually, only age >5 years, higher IL-10 level, higher IFN-γ level, and presence of complication entered the predictive model. Among the currently available predictive tools, a nomogram has high accuracy and good discrimination abilities in predicting outcomes with its convenience. The present nomogram performed well as supported by the C index values of 0.771 and 0.796 in the training and validation cohorts, respectively, and the optimal calibration curves demonstrating the agreements between prediction and actual observation.

A previous study showed that serum IL-18 and lactate dehydrogenase (LDH) levels can be used as parameters to determine which patients are candidates for corticosteroid therapy^19^. Serum LDH level might be a useful marker for the evaluation of therapeutic efficacy in refractory MPP^19^. A combination of age and serum levels of LDH, ESR ≥ 25 mm/h, and CRP ≥ 15 mg/L could be a predictive panel marker for early prediction of refractory MPP^18^. The serum concentrations of TNF-α and IFN-γ were significantly higher in children with refractory MPP^20^. Xu and colleagues demonstrated that age, fever duration, CRP and LDH were independent risk factors for BMP from patients with refractory MPP^5^. In our study, although serum CRP in MPP children with BMP was higher than those without BMP, its statistical power was relatively small and not included in our nomogram. Different from the previous studies, our study did not show a significant difference in serum LDH levels between the children with and without BMP. This result might be more convincing because of a much larger population in our study. On the other hand, due to the limited availability of the commercial cytometric bead array kit, our institute only detected IL-2, 4, 6, 10, TNF-α, and IFN-γ, other cytokines were not included in the present nomogram. Of course, combined detection of cytokines would have a greater confidence to predict the presence of BMP in children with MPP.

In our BMP risk estimation nomogram, age >5 years, higher IL-10 level, higher IFN-γ level, and presence of complication demonstrated significant predictive value. For clinical use of the model, we assessed the risk of BMP in children with MPP using 80 as the cutoff value. MPP children with a score of 80 or more are high-risk of BMP formation. For example, an MPP children with >5 years plus serum IFN-γ level >30 ng/L would have a total of 82 points (50 points for age >5 years, and 32 points for serum IFN-γ level), strongly indicating the BMP presence. In view of the prediction, the nomogram could serve as a tool to select patients for evaluating the necessity of flexible bronchoscopy. In general, the presence of persistent atelectasis in chest imaging is a strong indicator for flexible bronchoscopy intervention^27,28^. Thus early bronchoscopic examination may be necessary for children with MPP presented with clinical sign of atelectasis, regardless of the presence of BMP. Considering a strong drive of atelectasis for bronchoscopy intervention, atelectasis had the top score and the highest weight in our prediction nomogram. Notably, another complication of pleural effusion also showed similar value in the present predictive model. However, there was no relevant report about the relationship between pleural effusion and BMP formation in children with MPP. Their potential mechanism needs further investigation.

The use of the nomogram in estimating the risk of BMP in children with MPP to perform flexible bronchoscopy is a new concept. Our model depended on demographic data, thoracic imaging and serum cytokine levels to predict the risk of BMP in children with MPP. In addition, we demonstrated no improvement in model performance with the addition of variables originated from clinical manifestations (fever duration) and other laboratory markers (such as CRP level, neutrophil percentage, and serum IL-6 level). Although these variables are clearly important for diagnosis and management of MPP especially refractory MPP, the lack of improvement in model performance might further indicate complexity of MPP conditions^22,29,30^.

The present study had several limitations. First, our nomogram was internally validated using bootstrap methods, further studies are needed to externally validate the proposed nomogram. Moreover, a prospective study is required to further confirm the reliability of the nomogram. Second, because cytokine detections could not be available in all the patient or hospital, it would probably limit the popularity of this model. However, considering a higher weight of clinical variables for predicting BMP formation, clinicians should comprehensively assess a patient’s condition before making a decision. Also, data collection was limited to those children with 1 to 15 years, so it is not clear whether the model would apply to younger children (<1 year) or older children (>15 years). Third, in the present respective cohort, bronchoscopies were performed only in children with refractory MPP. Those children who got better after admission did not undergo bronchoscopy. The children could be considered to have no BMP. Therefore, it would inevitably lead to selective bias. Finally, because the nomogram was based on our institutional clinical data, other specific markers (such as IL-18) to estimate BMP risk might further improve the accuracy.

In conclusion, this study presents a nomogram demonstrating independent risk factors associated with BMP formation in children with MPP. The nomogram has potential to be used for early identification of BMP in children with MPP, contributing to a rational therapeutic choice.

## Supplementary information

Supplementary Information: R code.
