# Supplementary material for: Nomogram for Prediction of Bronchial Mucus Plugs in Children with Mycoplasma pneumoniae Pneumonia
Source: Sci Rep. 2020 Mar 12;10:4579. doi: 10.1038/s41598-020-61348-w (PMC7067858; doi:10.1038/s41598-020-61348-w)
Supplement: Supplementary file 1 — Supplementary Information: R code. [file 41598_2020_61348_MOESM1_ESM.pdf]

Nomogram for Prediction of Bronchial Mucus Plugs in Children with *Mycoplasma pneumoniae* Pneumonia

Xuefeng Xu, Huiwen Li, Yuanjian Sheng, Lei Wu, Danli Wang, Lingyue Liu, Yu Tong, and Zhimin Chen*

Department of Respiratory Medicine, Children's Hospital, Zhejiang University School of Medicine, National Clinical Research Center for Child Health, Hangzhou 310052, China

The R code

```
library(foreign)
library(rms)
library(mice)
attach(mp0)
#build a training model
train<-mp0[mp0$train=='1',]
train<-as.data.frame(train)
dd<-datadist(train)
options(datadist='dd')
fit1<-lrm(plug ~ age+complication2+IL102+IFN2, data=train, x=T, y=T)
fit1
summary(fit1)
nom1 <- nomogram (fit1, fun=plogis, fun.at=c(.001,.01,.05,
seq(.1,.9,by=.1) , .95, .99, .999),
                lp=F, funlabel="mucus plug")
plot(nom1)
nom1 <- nomogram(fit1, fun=plogis,fun.at=c(.1, .5,
                        seq(.2,.8, by=.1), .95, .99, .999),
                lp=T, funlabel="mucus plug")
plot(nom1)

cal1 <- calibrate(fit1, cmethod='hare',
                 method='boot', B=1000)
plot(cal1,xlim=c(0,1.0),ylim=c(0,1.0))
# build a testing model
test<-mp0[mp0$train=='0',]
test<-as.data.frame(test)
dd<-datadist(test)
options(datadist='dd')
fit2<-lrm(plug ~ age+complication2+
          IL102+IFN2,data=test,x=T,y=T)
fit2
summary(fit2)

nom2<- nomogram(fit2, fun=plogis,fun.at=c(.001, .01, .05,
                                     seq(.1,.9, by=.1), .95, .99, .999),
                lp=F, funlabel="mucus plug")
plot(nom2)
```

```
nom2 <- nomogram(fit2, fun=plogis,fun.at=c(.001, .01, .05, seq(.1,.9, by=.1), .95, .99, .999),
                  lp=T, funlabel="mucus plug")
plot(nom2)

cal2 <- calibrate(fit2, cmethod='hare',
                   method='boot', B=1000)
plot(cal2,xlim=c(0,1.0),ylim=c(0,1.0))


# calculate C statistics (equal to AUC)
library(ROCR)
train$predvalue<-predict(fit1)
pred <- prediction(train$predvalue,
                    train$plug)
perf<- performance(pred,"tpr","fpr")
perf
plot(perf)
abline(0,1)
auc <- performance(pred,"auc")
```
